# Comparison of different calculation techniques for absorbed dose assessment in patient specific peptide receptor radionuclide therapy

**DOI:** 10.1371/journal.pone.0236466

**Published:** 2020-08-07

**Authors:** Domenico Finocchiaro, Salvatore Berenato, Valentina Bertolini, Gastone Castellani, Nico Lanconelli, Annibale Versari, Emiliano Spezi, Mauro Iori, Federica Fioroni, Elisa Grassi

**Affiliations:** 1 Azienda Unità Sanitaria Locale di Reggio Emilia—IRCCS, Medical Physics Unit, Reggio Emilia, Italy; 2 Department of Physics and Astronomy, University of Bologna, Bologna, Italy; 3 Department of Medical Physics, Velindre Cancer Centre, Cardiff, United Kingdom; 4 Azienda Unità Sanitaria Locale di Reggio Emilia—IRCCS, Nuclear Medicine Unit, Reggio Emilia, Italy; 5 School of Engineering, Cardiff University, Cardiff, United Kingdom; National Institute of Health, UNITED STATES

## Abstract

**Aim:**

The present work concerns the comparison of the performances of three systems for dosimetry in RPT that use different techniques for absorbed dose calculation (organ-level dosimetry, voxel-level dose kernel convolution and Monte Carlo simulations). The aim was to assess the importance of the choice of the most adequate calculation modality, providing recommendations about the choice of the computation tool.

**Methods:**

The performances were evaluated both on phantoms and patients in a multi-level approach. Different phantoms filled with a ^177^Lu-radioactive solution were used: a homogeneous cylindrical phantom, a phantom with organ-shaped inserts and two cylindrical phantoms with inserts different for shape and volume. A total of 70 patients with NETs treated by PRRT with ^177^Lu-DOTATOC were retrospectively analysed.

**Results:**

The comparisons were performed mainly between the mean values of the absorbed dose in the regions of interest. A general better agreement was obtained between Dose kernel convolution and Monte Carlo simulations results rather than between either of these two and organ-level dosimetry, both for phantoms and patients. Phantoms measurements also showed the discrepancies mainly depend on the geometry of the inserts (e.g. shape and volume). For patients, differences were more pronounced than phantoms and higher inter/intra patient variability was observed.

**Conclusion:**

This study suggests that voxel-level techniques for dosimetry calculation are potentially more accurate and personalized than organ-level methods. In particular, a voxel-convolution method provides good results in a short time of calculation, while Monte Carlo based computation should be conducted with very fast calculation systems for a possible use in clinics, despite its intrinsic higher accuracy. Attention to the calculation modality is recommended in case of clinical regions of interest with irregular shape and far from spherical geometry, in which Monte Carlo seems to be more accurate than voxel-convolution methods.

## Introduction

Radiopharmaceutical therapy (RPT), as defined in ICRP 140 [1], is based on the use of specific pharmaceuticals labelled with radionuclides to deliver a lethal dose of radiation to tumour areas. Radiopharmaceuticals are specifically designed to have high affinity with given tumour sites, so that ionizing radiations, such as particles and photons emitted by the isotopes, may deposit energy inside or close to unhealthy tissues, saving surrounding healthy tissues. This approach produced very encouraging results in the treatment of neuroendocrine tumours (NET) in the last decades, in particular in therapies which make use of somatostatin analogues labelled with 90Y or 177Lu [2], such as the recently registered Lutathera [3]. Different response rates and a large inter-patient variability of the outcome were however reported by some authors (e.g. Campana D and Vinjamuri S [4, 5]).

The well-established experience with external beam radiation therapy (EBRT) has provided strong evidence that tumour response and normal organ toxicity is related to absorbed doses. For this reason it was supposed that the treatment outcome correlates with the absorbed dose delivered to tumours even in RPT [6, 7]. Yet, a dosimetry as more accurate and personalized as possible is needed to this purpose, to provide clinicians with reliable results.

Despite the general demand for a more individualized treatment based on pre-therapeutic dosimetry study in NET, dosimetry is not conducted always in the clinical routine. This is mostly because dosimetry is often considered time consuming (a lot of time required for imaging), expensive (costs for every image scan and every measurement) and sometimes inaccurate (for the lack of standardization and harmonization mainly). At present a standard procedure for calculating the absorbed dose is not well defined for every kind of radionuclide therapy. In relation to NET, the evidence of prolonged survival has been demonstrated only recently [3] in a subgroup of NET.

Different methods have been developed to perform dosimetry since its beginnings. Techniques based on standardized reference models were first developed thanks to their simplicity of implementation and have been used for many years. These models assume uniform activity (i.e. homogeneous uptake) in the source regions. However, evidence indicates that deterministic biological effects including tumour response and normal tissue toxicity may not be well predicted by the mean absorbed dose in the region and may be significantly influenced by non-uniform doses [8]. To take into account this aspect, voxel-based techniques were considered, similarly to those which have been also used for decades as standard of care in EBRT [9, 10]. Contrary to what happens for EBRT, however, in which plenty of software for therapy planning are available on the market, in RPT only few systems, which are adequate to dosimetry for Peptide Receptor Radionuclide Therapy (PRRT) and which can work with multiple 3D imaging, have been officially released in the last few years [11–13]. For this reason, many dosimetry software and tools are in use worldwide, but only some of them are commercially available. Several of them are home-made tools, which were developed before the commercial software were finalized [14–20] and have been fully customized by clinical users in the meantime.

At present standardization and harmonization of the calculation systems are important. Therefore, it is essential to compare the various results obtained with the most advanced existing home-made/commercial software and other less advanced still used worldwide methods. Yet, both categories should be tested on a larger sample of cases than ever done before. These tests should provide an example of the most accurate methodology for 3D dosimetry in RPT, thanks to the gained experience in the last decades, giving recommendations about the appropriate use and the limitations of each method.

A few studies presenting some comparisons have already been published [18–20]. However, these works either did not report dosimetry studies performed completely at the voxel level [18], or considered a limited number of clinical cases [19], or showed a comparison based on the dose factors and not based on absorbed doses [20]. Therefore, more studies are needed to fully evaluate calculation performances in clinically relevant conditions, considering a high number of cases.

In this context, the main objective of the present work is to compare different modalities for absorbed dose calculation, to point out the pros and the cons in each modality, and to provide recommendations about the choice of the most adequate computation technique for the single clinical or research centres approaching the methodology. The modalities here considered include the most used techniques in this field worldwide, ranging from the less advanced and less personalised, to the most accurate and patient specific.

The considered modalities listed in growing complexity are: organ level dosimetry based on standardized reference models (such as OLINDA version 1.1 [21], which has been used for decades before the recent release of the new updated commercial version OLINDA version 2 [22]), voxel-level dosimetry based on dose kernel convolution (VoxelMed2.0 [23]), and voxel level dosimetry based on Monte Carlo (MC) simulations (RAYDOSE [24]). OLINDA1.1 was chosen because it is still widely used for RPT dosimetry. VoxelMed2.0 was chosen because it was designed to achieve a good compromise between calculation accuracy and easy applicability in clinical practice. RAYDOSE was considered because MC techniques are considered to provide the most accurate approach to dose estimate [25].

The comparison was performed on 3D images of specifically designed phantoms and on multiple 3D dataset of images of a high number of clinical cases. This multi-approach method based both on phantoms and patients allowed to investigate the differences of performance between the calculation modalities (depending on the shape and the volume of the activity distribution) and to provide a valuable comparison based on a conspicuous number of clinical cases.

## Materials and methods

This study involves human participants. All participants were enrolled in a clinical trial (EUDRACT 2013-002605-65) at Azienda USL-IRCCS of Reggio Emilia (Italy) The study was approved by the ethics committee of Azienda USL-IRCCS of Reggio Emilia (Italy) and each patient gave written informed consent for the study conduction.

The following sections describe in detail the specific phantoms, the image set, the software and the data elaboration approach.

### Preparation of phantoms

**Fig 1:**
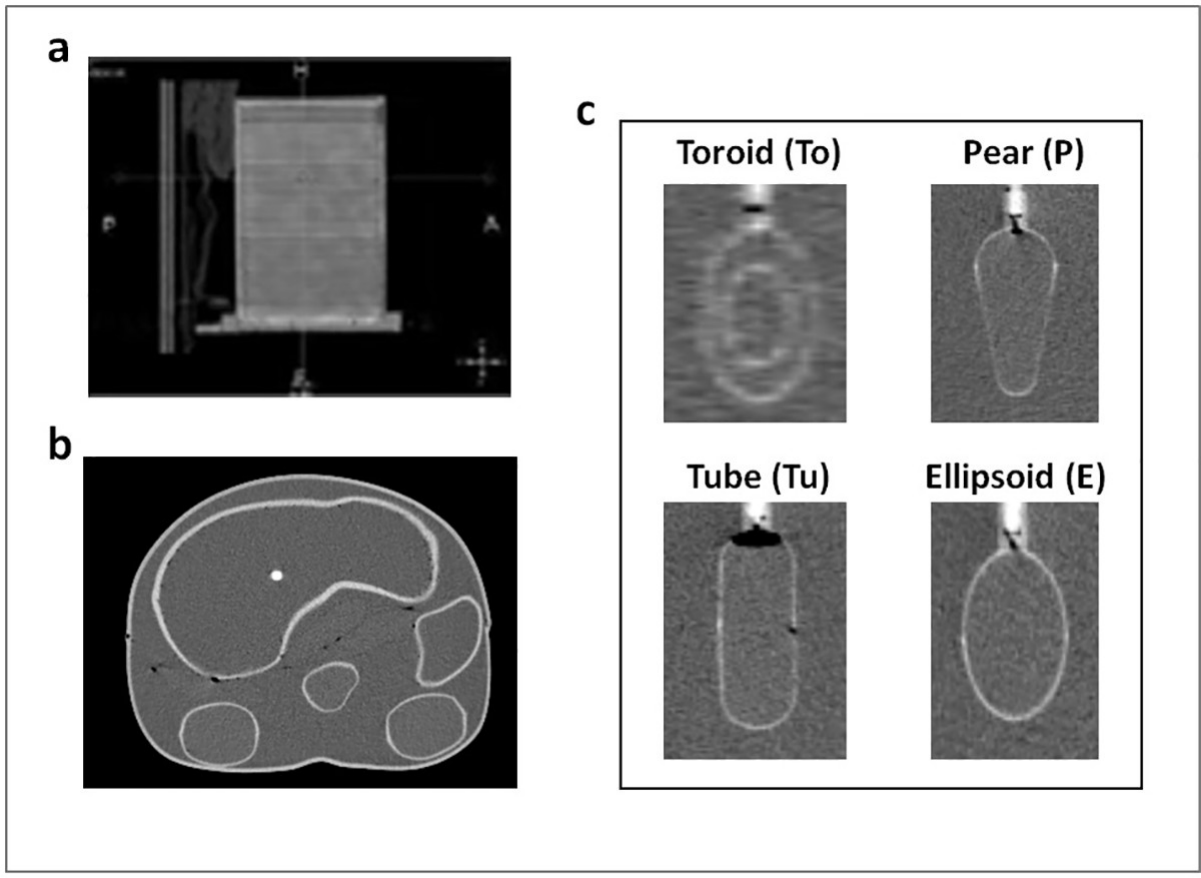
CT scans of phantoms used in this study. (a) Cylindrical phantom. (b) Anthropomorphic phantom. (c) Inserts with different shapes placed in the Geometrical phantom.

Three different phantoms filled with ^177^Lu radio-labelled peptides left-over from the clinical application were used:

- a ‘Cylindrical phantom’ filled with a homogeneous radioactive solution (Jaszczak, Data Spectrum Corporation; USA) shown in Fig 1A. Details are included in Table 1.
- a cylindrical phantom and a set of 11 fillable plastic inserts arranged in two different configurations, to originate a couple of ‘Geometrical phantoms’. The inserts, different for shape (toroidal, pear-shaped, tubular and ellipsoidal) and volume, are depicted in Fig 1C. Inserts take the name from the shape and the equivalent diameter (i.e. the diameter for a sphere with the same volume), as shown in Table 2. Each insert was filled with the same activity concentration and placed in a non-radioactive water background. Details of volume and activity concentration are shown in Table 1.
- an ‘Anthropomorphic phantom’ with organ shaped inserts (Liqui-Phill, The Phantom Laboratory, Greenwich, NY) shown in Fig 1B. Details are included in Table 1. Every insert was filled with an activity concentration typical of real organs in clinical cases, and placed in a radioactive water background.

**Table 1:** Description of phantoms used to test the dosimetry tools.

Phantom	Phantom volume (ml)	Insert name	Insert volume (ml)	Insert activity concentration (MBq/ml)	Background activity concentration (MBq/ml)
**Cylindrical**	5640	N/A	N/A	0.25	N/A
**Geometrical**	6713	To17a	2.8	1.53	N/A
		To26	9.7		
		E20	4.1		
		E30	14.8		
		E38	28.5		
		To17b	2.8	1.53	
	6713	P38	29.2		
		P39a	30.1		
		P39b	31.2		
		Tu38a	28.6		
		Tu38b	28.8		
**Anthropomorphic**	11600	Lesion	2.0	8.34	0.03
		Pancreas	92	0.99	
		Left kidney	142	0.81	
		Right kidney	142	0.82	
		Spleen	156	1.10	
		Liver	1470	0.53	

**Table 2:** Legend of the insert acronyms for the Geometrical phantom.

Insert geometry	Equivalent diameter (mm)	Insert name
**Torus**	17	To17a
**Torus**	17	To17b
**Torus**	26	To26
**Ellipsoid**	20	E20
**Ellipsoid**	30	E30
**Ellipsoid**	38	E38
**Pear**	38	P38
**Pear**	39	P39a
**Pear**	39	P39b
**Tube**	38	Tu38a
**Tube**	38	Tu39b

To accurately measure the volumes, the weight of the phantoms and of the inserts (before and after refilling) was taken with a calibrated scale. The density of the water-based solution was of 1g/ml. HCl (0.1 M) was used as a carrier solution to prevent radioactive ^177^Lu deposition on the phantom walls and to guarantee a homogenous radionuclide solution.

Every phantom was scanned once and the time-activity curve was generated using the physical decay of the isotope. All specific data regarding the volumes of inserts and phantoms, and the activity used are reported in Table 1.

### Clinical trial

The clinical cases considered in the present work were all extracted from a pre-existing clinical PRRT trial including 100 patients and conducted by Azienda USL-IRCCS of Reggio Emilia (Italy).

All considered patients were previously enrolled in the trial (EUDRACT 2013-002605-65) between 2014 and 2015. The clinical trial design established that every patient had to be sequentially administered with either ^177^Lu labelled radiopeptides (^177^Lu-DOTATOC) or ^90^Y labelled radiopeptides (^90^Y-DOTATOC), up to a maximum of 5 infusions (or cycles). Dosimetry was mandatory in the clinical trial and was to schedule during the first cycle of therapy after a therapeutic administration of 177Lu-DOTATOC. Each patient underwent 5 SPECT/CT scans at 1, 4, 24, 44, 72 h post injection. According to the trial design, clinical absorbed doses for ^177^Lu and ^90^Y labelled radio-peptides for liver, spleen and kidneys were calculated. Each organ was manually contoured and absorbed doses were calculated in compliance with the MIRD scheme [26] at organ-level from images. The number of cycles, the isotope and the activity chosen for every injection were planned by an expert physician, on the basis of the dosimetry results. The activity prescription had to be determined based on the Biological Effective Dose (BED) delivered to kidneys. Kidneys are regarded as the principal organs at risk in PRRT [27, 28]. As suggested by different works [29–31], in this clinical trial the cumulative dose limit to kidneys was set to 46 Gy of BED for patients with no risk factors (hypertension, diabetes, renal failure are considered risk factors for this therapy) and at 28Gy for patients with risk factors.

In the present work absorbed doses to kidneys, spleen and liver were calculated to compare the three dosimetric methods.

### Image acquisition and reconstruction

All activity measurements were performed with an accurate activity calibrator for ^177^Lu (Aktivimeter Isomed 1010, Nuklear Medizintechnik, Germany) and all image acquisitions were performed through a SPECT-CT scanner (Symbia T2, Siemens Medical, Germany, 3/8” NaI(Tl)-detector) previously calibrated [23]. The standard clinical protocol for body studies was used both for phantoms and patients with the following SPECT settings: MEHR collimators; matrix 128 x 128; zoom = 1; views = 32 x 2; time/view = 30 s; step and shoot mode; degree of rotation = 180°; non-circular orbit; detector configuration = 180°.

The first CT acquisition per patient was performed with the following parameters: 130kV and max = 90 mAs using tube current modulation. The subsequent CT images were acquired with 130 kV and 40mAs for radiation protection safety of patients. The CT reconstructed slice thickness was 5 mm and a smooth reconstruction kernel was used (B08s; Siemens Medical Solution, Germany). The higher image quality of the first CT scan is necessary for contouring volumes of interest more accurately.

The SPECT projections were reconstructed by an iterative algorithm including CT attenuation correction, scatter correction and full collimator-detector response in Siemens E-Soft workstation (Syngo, MI Application version 32B, Siemens Medical Solution, Germany) with Flash 3D iterative algorithm (10 iterations; 8 subsets; Gaussian filter cut-off = 4.8 mm; 4.8 mm cubic voxel) [32].

All cases of Sample A were rigidly registered to the first CT image of the sequence in Siemens E-soft workstation. Images of patients included in Sample B were registered using a deformable multi-pass algorithm with the Velocity Advanced Imaging workstation 3.2.0 (Varian Medical Systems, Palo Alto, USA) [33]. The registration procedures rescaled the original voxel size to 3.9x3.9x3.5 mm^3^.

The Volumes Of Interest (VOI) for each phantom and each patient were manually drawn on the reference CT image, as recommended by Uribe et al [34], using the Velocity workstation.

### Software for image processing and dosimetry calculations

#### OLINDA1.1

OLINDA version 1.1 [21] is an organ level dosimetry software based on the MIRD methodology [26] for internal dose estimation. This is the method adopted in the clinical trial the clinical cases of this work are extracted from. Absorbed doses to organs and to lesions can be calculated by using different models in the software: human phantom models, i.e. mathematical representations of the human body to represent organs and whole body, and sphere models, i.e. mathematical representations of spheres to represent lesions [35, 36].

Unlike VoxelMed and RAYDOSE, OLINDA needs time-integrated activity $\tilde{A}$ values of VOIs as input parameters [37], which were calculated with VoxelMed2.0 (which will be described in the next section) and then inserted in OLINDA.

OLINDA sphere model (commonly used to calculate doses to lesions) was used to generate the results for the inserts placed in the Geometrical phantom and for the dummy lesion housed in the anthropomorphic phantom, while OLINDA organ model (adult male) was used for the dummy organs placed in the anthropomorphic phantom. Real insert volumes were used for calculations.

The human models (adult male or adult female) were used to calculate dosimetry of the cohort of patients. Doses were scaled using the true patient weight and the true organ masses.

#### VoxelMed2.0

VoxelMed is a home-made software for dose calculation developed at Azienda USL-IRCCS research hospital (Reggio Emilia, Italy). It was developed in the Matlab (The Mathworks, Natick, MA) programming environment and designed on the CERR platform (www.cerr.info). It performs voxel-level dosimetry based on the MIRD guidelines [38]. The first version of the software along with the S value matrices for voxel dosimetry used in calculations were described in detail elsewhere [23].

VoxelMed version 2.0 includes a graphical user interface, the possibility to export the results of calculations to Microsoft Excel file, the visualization of the fitting curves (both mono- and bi-exponential), a module for renal BED calculation following the model suggested by Strigari L et al. [39] and the possibility to correct activity for partial volume effect (PVE), as presented in [40]. Moreover VoxelMed2.0 provides the user with the time-integrated activity $\tilde{A}$ at VOI level, which can be used for dosimetry with OLINDA version 1.1 both for organs and lesions.

To calculate the number of disintegrations VoxelMed integrates the time-activity curve with the trapezoidal method in the time interval between the first and the last acquisition. Beyond this time-interval the integration is performed analytically, and the time-activity curve is extrapolated using the effective half-life or the physical half-life (it is chosen by the user). The effective half-life of the organ or lesion is derived with a bi-exponential fit of the activities in the VOI, the physical half-life is known from the selected isotope. Time-integrated activity is calculated in each voxel or in the whole organ depending on the modality of dose calculation selected (i.e. voxel level or organ level).

#### RAYDOSE

RAYDOSE is a software package developed at Cardiff University (School of Engineering, Cardiff University, UK) and designed to carry out 3D patient-specific image-based dosimetry for RPT. RAYDOSE provides personalized 3D dose map performing Monte Carlo simulations on radiation transport based on the Geant4 MC toolkit (CERN, Switzerland). Geant4 is the state-of-the-art package for the simulation of the transport of particles through matter [41]. RAYDOSE generates voxel-level dose maps using anatomical and physiological data taken from morphologic and functional images [24].

In order to obtain the area under the time-activity curve, RAYDOSE allows to use different fitting modalities: mono-exponential decay, linear uptake plus mono-exponential decay or the trapezoidal method. In this study, for the dose calculation of the clinical cases, we used the trapezoidal method at the voxel level up to the last time acquisition point, while the time-activity curve beyond the last scan time was extrapolated from the mono-exponential curve fitting of the whole organ activities in the VOI. For dose calculation in phantoms, we used the physical half-life of the isotope to extrapolate the activity from the scan time upwards.

### Data and statistical analysis

Two groups of patients were considered for the purpose of this work.

A first subgroup of 50 cases (named as “Sample A”) was extracted by random sampling from the original clinical trial to adequately represent the whole population. The number of cases was calculated safely adopting a margin of error of 10% and a standard deviation of 50%.

A second independent subgroup of 20 patients (named as "Sample B") was extracted too from the original clinical trial, similarly to Sample A. A sample of 20 cases was considered adequate in relation to the aim of the experiment conducted on Sample B.

Patient baseline characteristics for Sample A and Sample B are reported in Table 3.

**Table 3:** Demographic and baselines clinical characteristics of all patients*.

Characteristic	Sample A (N = 50)	Sample B (N = 20)
**Gender (No)**		
** Male**	28 (56.0%)	9 (45.0%)
** Female**	22 (44.0%)	11 (55.0%)
**Age (y)**	60 ± 12	62 ± 12
**Height (cm)**	168 ± 9	166 ± 8
**Weight (kg)**	73 ± 15	68 ± 14
**Primary tumour site (No)**		
** Ileum**	18 (36.0%)	7 (35.0%)
** Pancreas**	8 (16.0%)	6 (30.0%)
** Lung**	5 (10.0%)	N\A
** Thyroid**	3 (6.0%)	N\A
** Rectum**	2 (4.0%)	N\A
** Others**	14 (28.0%)	7 (35.0%)
^**177**^**Lu activity for dosimetry (MBq)** †	4440 ± 1000	± 857

* Plus-minus values are means ± standard deviation.

† Injected activity at the first cycle of therapy. Dosimetry was performed after the first injection.

The study type, the image registration and the software used for the dose calculations of the clinical cases in sample A and sample B are summarised in Table 4.

**Table 4:** Summary of phantom and patient studies performed.

Study type	Object of study	Image registration	Software
**Phantom**	Homogeneous phantom	No registration (only 1 scan)	OLINDA1.1—VoxelMed—RAYDOSE
	Geometrical phantom	No registration (only 1 scan)	OLINDA1.1—VoxelMed—RAYDOSE
	Anthropomorphic phantom	No registration (only 1 scan)	OLINDA1.1—VoxelMed—RAYDOSE
**Clinical**	Sample A (50 patients)	Rigid registration	OLINDA1.1—VoxelMed
	Sample B (20 patients)	Deformable registration	OLINDA1.1—VoxelMed VoxelMed^(λ RD)^—RAYDOSE

Absorbed doses were calculated separately with OLINDA1.1, VoxelMed2.0 and RAYDOSE using the same set of images.

**Fig 2:**
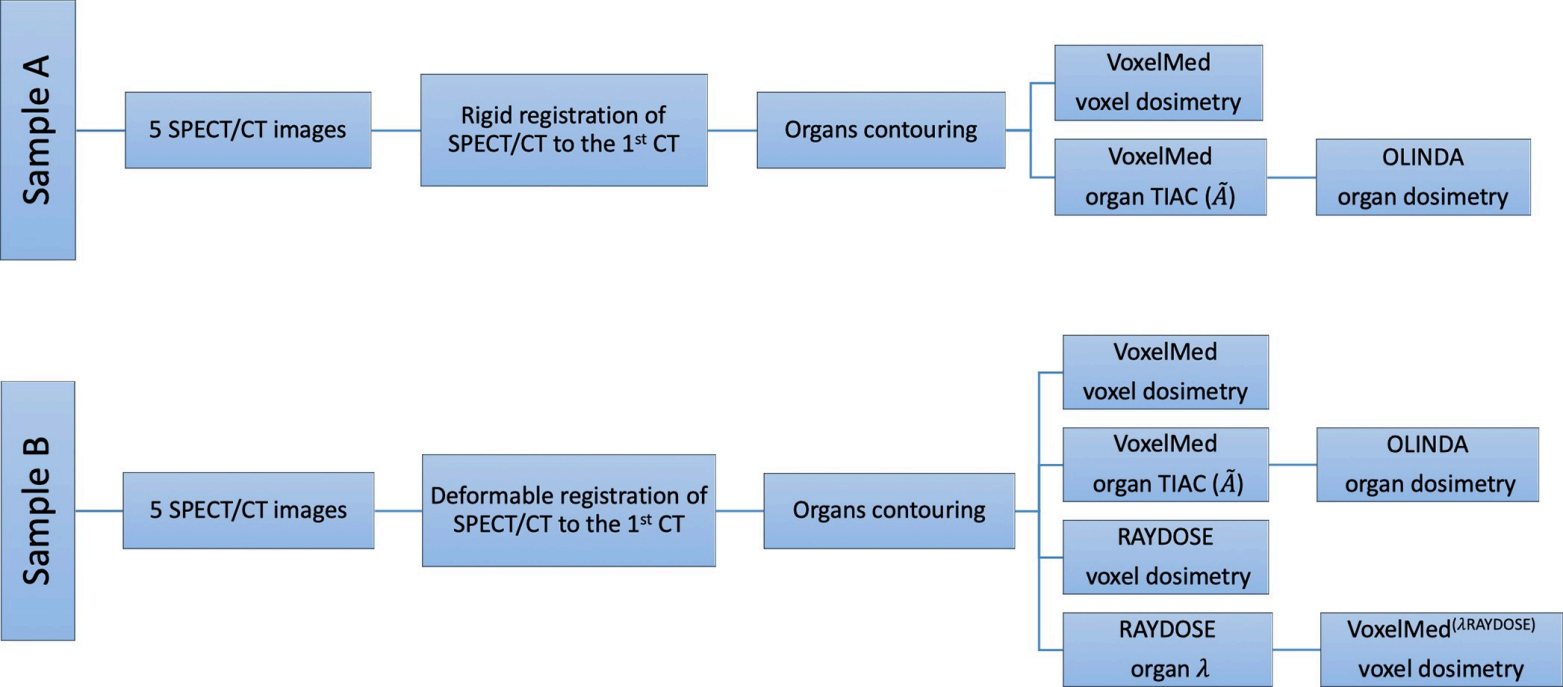
Clinical comparison study workflow. Procedure flow of absorbed dose calculation for each patient of sample A (top of the image) and sample B (lower part of the image).

Kidney, liver and spleen absorbed doses were calculated for each patient. Two different comparison studies were performed. The first study involved only comparison between VoxelMed and OLINDA based on absorbed dose calculations of patients in Sample A. The second study involved comparison between all the three software (VoxelMed, OLINDA and RAYDOSE) based on absorbed dose calculations of patients in Sample B. Furthermore, in order to reduce the contribution of the fitting of the activity-time curves in the comparison of the software, the VoxelMed dosimetry calculations for Sample B were repeated using the same effective half-life applied in RAYDOSE. RAYDOSE estimates the effective half-life by fitting the organ activities against time, as previously described. Flow chart in Fig 2 illustrates methodology in clinical study.

Dose-volume histograms (DVH) were evaluated to compare spatial dose distribution at voxel-level.

Mean values of absorbed dose were used to compare organ level and voxel level techniques.

Comparison between the different dosimetry methods was statistically evaluated using the Lin’s concordance correlation coefficient (CCC) and the Bland-Altman plot [42]. The CCC, symbolized by ρ_c_, allows to evaluate the degree of concordance between two measures, while the Bland-Altman plot is used to analyse the agreement between two quantities. The CCC was calculated using SAS 9.3 (SAS Institute, Cary, NC, USA). A value of ρ_c_ equal to +1 denotes perfect concordance, a value equal to -1 perfect discordance, while a value of 0 no correlation.

## Results

### Physical phantom study

**Fig 3:**
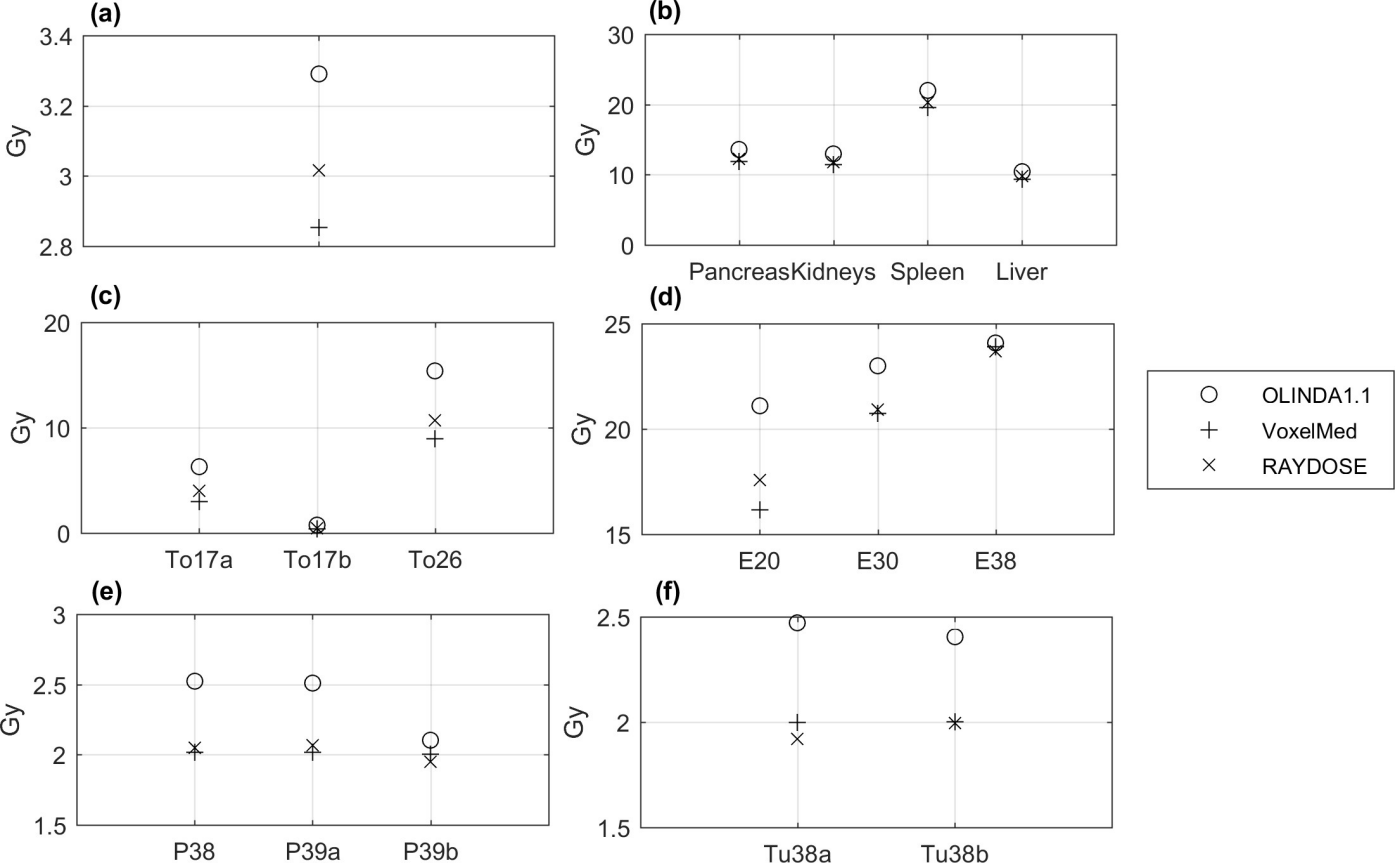
Comparison of mean absorbed dose (Gy) calculated using OLINDA1.1, VoxelMed and RAYDOSE. (a) Homogeneous phantom, (b) Anthropomorphic phantom and (c-f) Geometrical phantom. Note the Lesion insert in the Anthropomorphic phantom is missing because beyond the range of dose visualized in the graph.

The values of mean absorbed dose for the physical phantoms calculated with OLINDA1.1, VoxelMed and RAYDOSE are reported in Table 5. Visual representation of the same data is provided in Fig 3.

**Table 5:** Mean absorbed dose (Gy) calculated with OLINDA1.1, VoxelMed and RAYDOSE for all of the three phantoms. The absorbed dose calculated with OLINDA1.1 was performed using either the Organ model and the Sphere model. Absorbed doses to Pancreas, Kidneys, Spleen and Liver were calculated using the Organ model, otherwise the Sphere model was used.

Phantom	Insert name	OLINDA1.1	VoxelMed	RAYDOSE
**Cylindrical**	N/A	3.3	2.8	3.0
**Geometrical**	To17a	6.3	3.0	4.0
	To17b	0.8	0.4	0.5
	To26	15.4	9.0	10.7
	E20	21.1	16.1	17.6
	E30	23.0	20.7	20.9
	E38	24.1	23.9	23,7
	P38	2.5	2.0	2.1
	P39a	2.5	2.0	2.1
	P39b	2.1	2.0	2.0
	Tu38a	2.5	2.0	1.9
	Tu39b	2.4	2.0	2.0
**Anthropomorphic**	Lesion	102.3	91.3	97.9
	Pancreas	13.6	11.9	12.2
	Kidneys	12.9	11.4	11.8
	Spleen	22.0	19.6	20.3
	Liver	10.4	9.3	9.8

**Fig 4:**
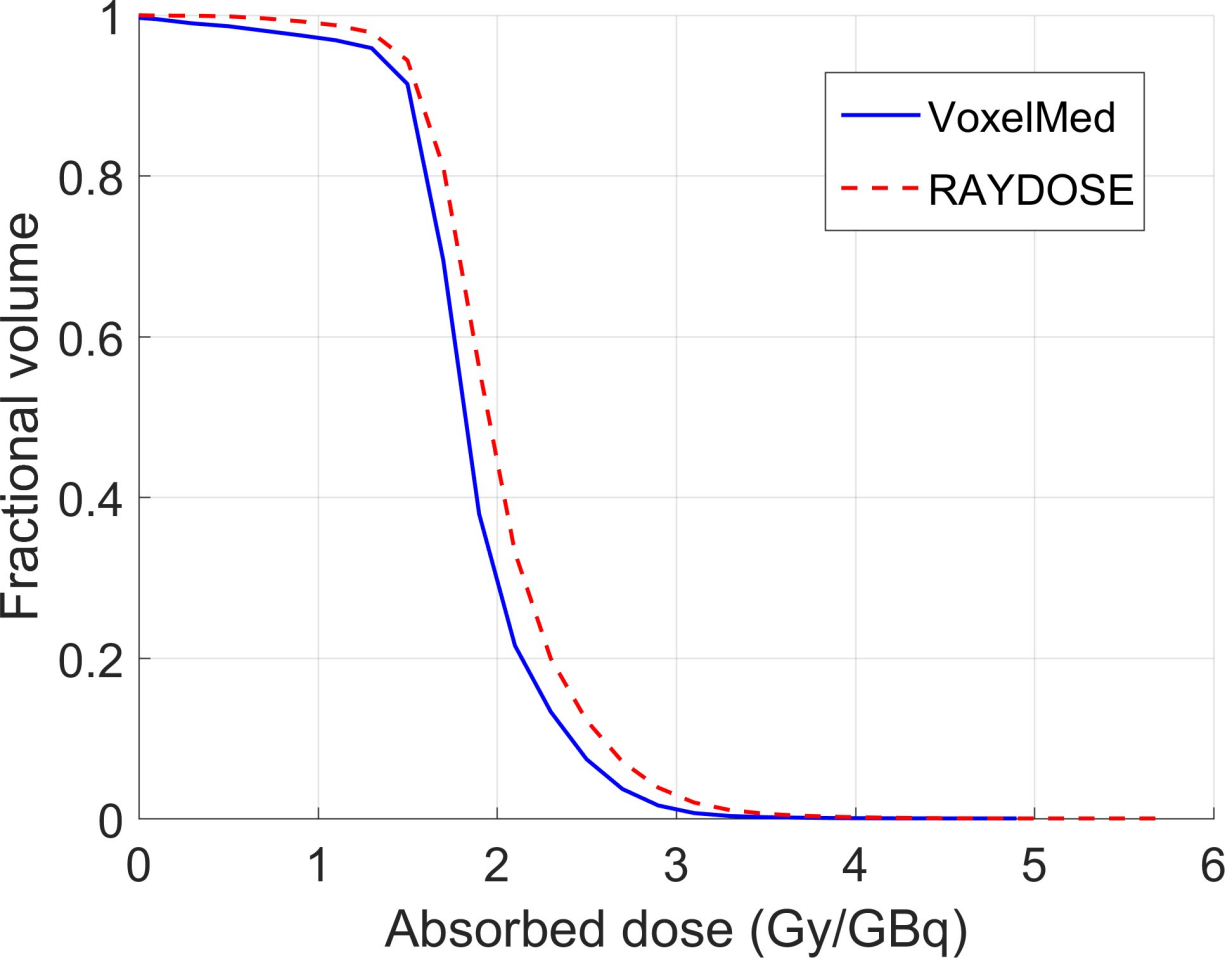
Comparison of DVHs calculated using VoxelMed (continuous line) and RAYDOSE (dotted line) for the Cylindrical phantom.

**Fig 5:**
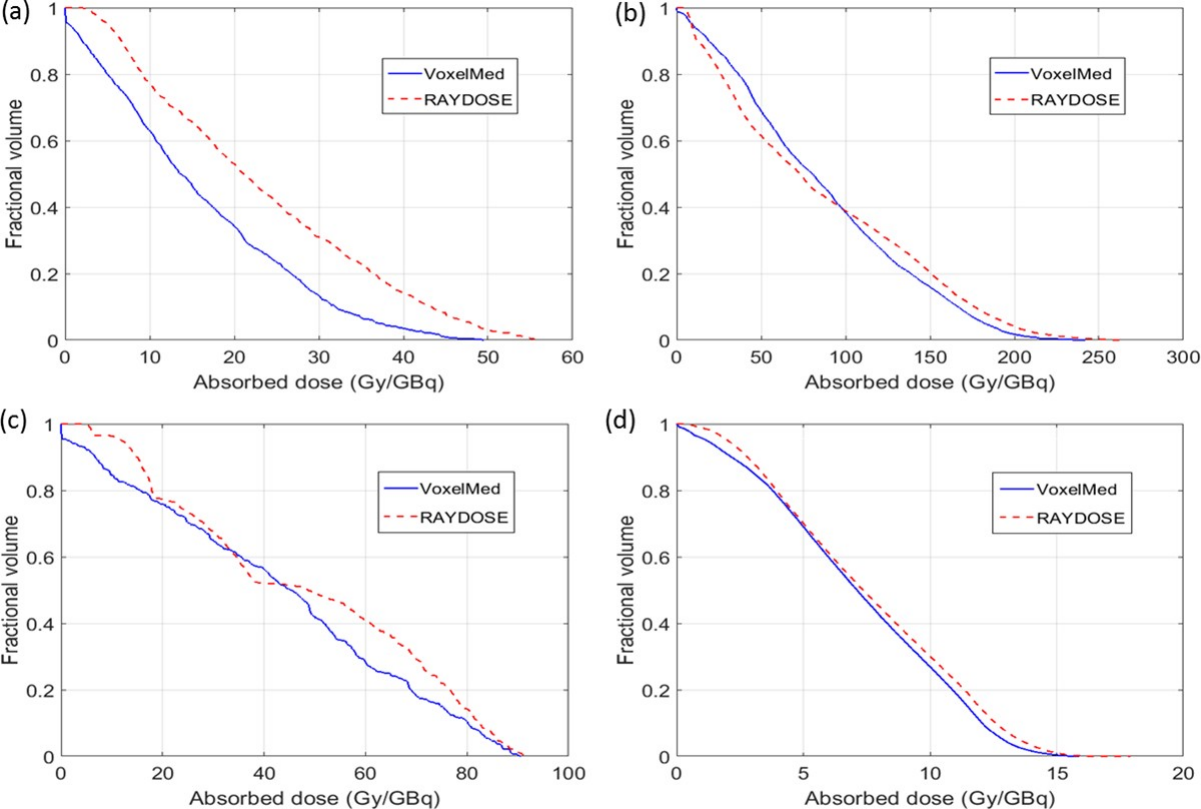
Comparison of DVHs calculated using VoxelMed (continuous line) and RAYDOSE (dotted line). Several inserts were considered: Geometrical phantom inserts (a) To17a and (b) TU38b; Anthropomorphic phantom inserts (c) Lesion and (d) Right Kidney.

Similar DVH curves were generated with VoxelMed and RAYDOSE both for the Cylindrical phantom (Fig 4) and for the other two phantoms (Fig 5). Fig 5 shows DHVs only for the inserts in the Geometrical and the Anthropomorphic phantoms with the smallest and the largest relative differences of mean absorbed dose, respectively.

### Clinical study

**Fig 6:**
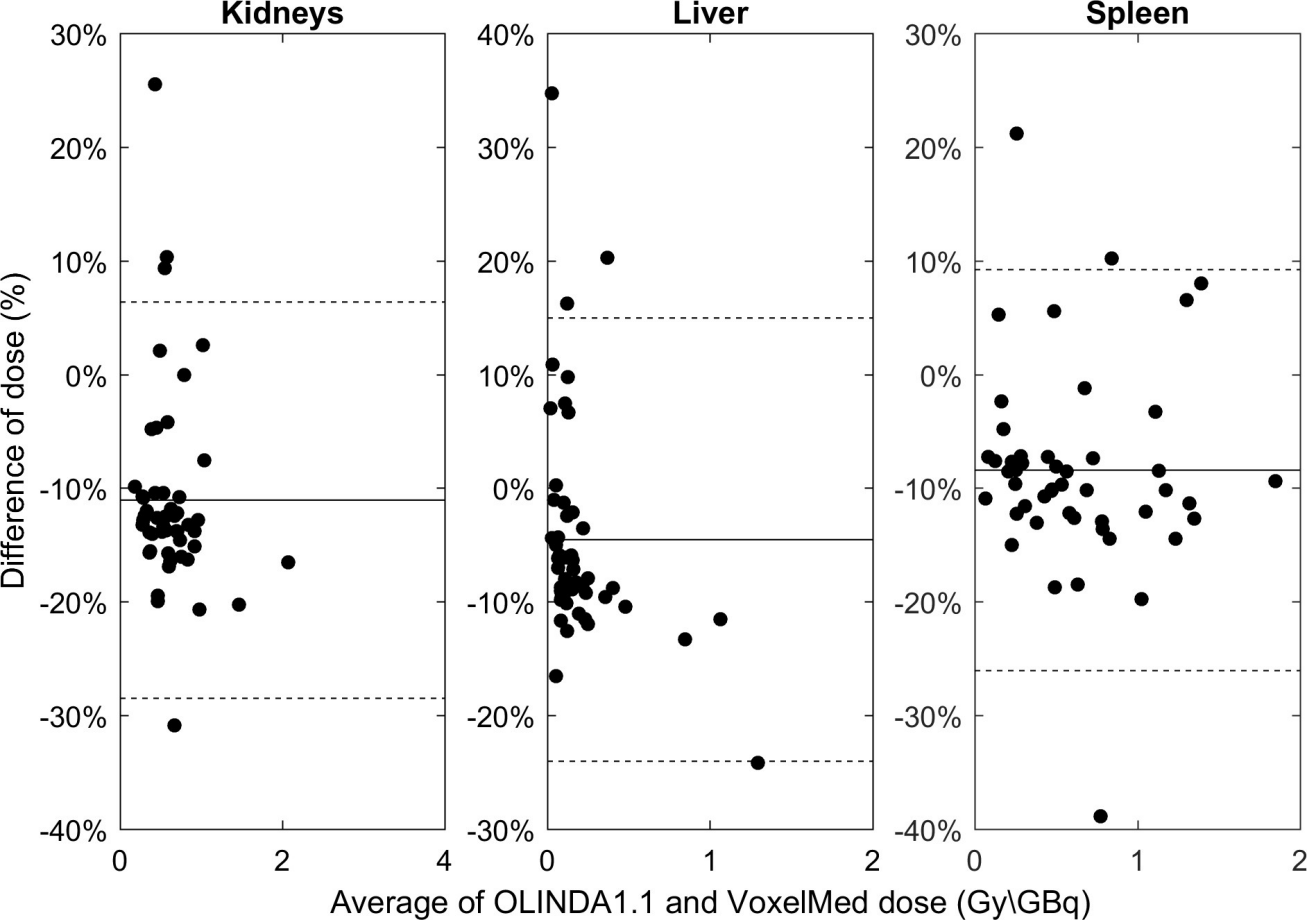
Comparison of mean absorbed dose per unit of injected activity (Gy/GBq) calculated using VoxelMed and OLINDA1.1. A Bland-Altman plot was used to compare the calculated absorbed dose in kidneys, liver and spleen of patients in Sample A. The percentage difference between VoxelMed compared to OLINDA1.1 absorbed dose was calculated. Dashed lines are the limits of agreement (±1.96 SD), while the horizontal solid lines are the average percentage differences.

Absorbed dose for kidneys, liver and spleen of the sample A of patients calculated with OLINDA1.1 and VoxelMed are shown in Table 6. The absorbed doses to liver and to spleen were found to be highly correlated, while lower correlation was found for kidneys. The CCC [95% confidence interval] values were ρ_c, liver_ = 0.97 [0.96, 0.98], ρ_c, spleen_ = 0.85 [0.76, 0.92], ρ_c, kidneys_ = 0.55 [0.39, 0.68]. The Bland-Altman plot is shown in Fig 6.

**Table 6:** Mean absorbed dose (Gy/GBq) calculated with OLINDA1.1 and VoxelMed for kidneys, liver and spleen for patients of Sample A.

Cases	Kidneys		Liver		Spleen	
	OLINDA1.1	VoxelMed	OLINDA1.1	VoxelMed	OLINDA1.1	VoxelMed
**1**	0.84	0.99	0.13	0.11	1.34	1.26
**2**	0.69	0.83	0.08	0.09	0.57	0.70
**3**	0.42	0.52	0.15	0.16	0.28	0.30
**4**	0.44	0.46	N/A	N/A	0.21	0.25
**5**	0.56	0.67	0.38	0.42	0.06	0.07
**6**	0.33	0.39	1.11	1.47	0.24	0.27
**7**	0.57	0.59	0.13	0.12	0.88	0.80
**8**	0.42	0.52	0.05	0.05	0.20	0.22
**9**	0.79	0.79	0.06	0.07	0.48	0.52
**10**	1.30	1.63	0.23	0.27	0.91	1.14
**11**	0.76	0.91	0.40	0.33	0.59	0.96
**12**	0.55	0.80	0.15	0.16	0.27	0.30
**13**	0.43	0.49	0.04	0.04	0.15	0.14
**14**	0.33	0.40	0.08	0.09	N/A	N/A
**15**	0.66	0.75	0.17	0.18	1.08	1.18
**16**	0.27	0.31	0.03	0.03	0.17	0.18
**17**	1.89	2.26	0.45	0.51	1.26	1.44
**18**	0.78	0.90	0.18	0.21	0.98	1.12
**19**	0.90	1.03	0.15	0.17	1.09	1.13
**20**	0.68	0.80	0.08	0.09	0.76	0.89
**21**	0.47	0.55	0.20	0.22	0.41	0.45
**22**	0.55	0.66	0.24	0.26	0.73	0.85
**23**	0.57	0.53	0.22	0.25	1.14	1.33
**24**	0.53	0.61	0.11	0.12	0.45	0.50
**25**	1.04	1.01	0.14	0.15	0.65	0.72
**26**	0.54	0.65	0.11	0.11	0.54	0.59
**27**	0.27	0.31	0.08	0.09	N/A	N/A
**28**	0.37	0.43	0.11	0.13	0.57	0.65
**29**	0.50	0.49	0.06	0.07	0.50	0.48
**30**	1.00	1.08	0.10	0.10	0.67	0.68
**31**	0.38	0.40	0.22	0.22	0.16	0.17
**32**	0.34	0.40	1.00	1.13	0.44	0.54
**33**	0.31	0.35	0.12	0.12	0.22	0.24
**34**	0.48	0.39	0.13	0.13	0.28	0.23
**35**	0.26	0.30	0.02	0.02	0.08	0.09
**36**	0.64	0.75	0.11	0.12	0.73	0.84
**37**	0.62	0.71	0.34	0.37	1.76	1.94
**38**	0.50	0.56	0.11	0.12	0.55	0.62
**39**	0.85	0.99	0.14	0.16	1.24	1.40
**40**	0.59	0.67	0.06	0.07	0.51	0.56
**41**	0.29	0.33	0.79	0.91	0.24	0.26
**42**	0.52	0.60	0.13	0.14	0.43	0.47
**43**	0.27	0.30	0.03	0.03	0.12	0.13
**44**	0.18	0.20	0.23	0.25	0.45	0.50
**45**	0.86	1.09	0.05	0.06	0.35	0.41
**46**	0.50	0.58	0.08	0.09	0.29	0.33
**47**	0.40	0.45	0.05	0.06	0.70	0.76
**48**	0.69	0.78	0.11	0.12	1.11	1.24
**49**	0.60	0.55	0.07	0.08	1.45	1.34
**50**	0.65	0.76	0.03	0.02	0.23	0.26

**Fig 7:**
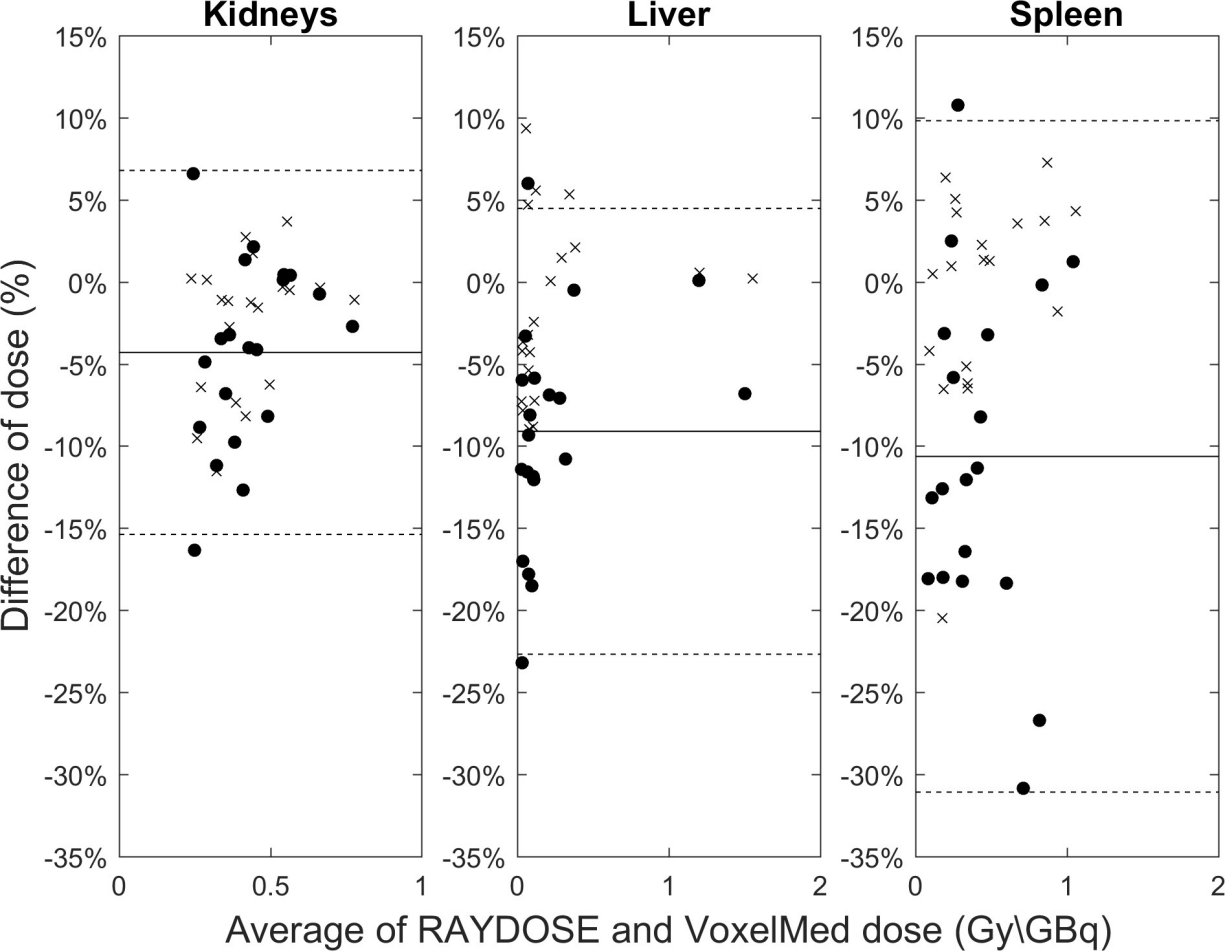
Comparison of mean absorbed dose per unit of injected activity (Gy/GBq) calculated using RAYDOSE, VoxelMed and VoxelMed ^(λ RD)^. A Bland-Altman plot was used to compare the calculated absorbed dose in kidneys, liver and spleen doses of patients in Sample B. The percentage difference between RAYDOSE compared to VoxelMed (dot) or VoxelMed ^(λ RD)^ (cross) absorbed dose was calculated. The limits of agreement (dashed line), corresponding to ±1.96 SD, were calculated based on the difference between VoxelMed and RAYDOSE.

OLINDA1.1, VoxelMed, VoxelMed^(λ RD)^ and RAYDOSE calculated mean absorbed dose for patients of Sample B are shown in Table 7, while the Bland-Altman plot is shown in Fig 7. The absorbed doses calculated with VoxelMed and RAYDOSE were highly correlated, with ρ_c, kidneys_ = 0.98 [0.95, 0.99], ρ_c, liver_ = 0.99 [0.99, 1.00], and ρ_c, spleen_ = 0.94 [0.87, 0.97], and almost complete agreement were found between VoxelMed^(λ RD)^ and RAYDOSE, with ρ_c, kidneys_ = 0.99 [0.98, 1.00], ρ_c, liver_ = 1.00 [1.00, 1.00], and ρ_c, spleen_ = 1.00 [0.99, 1.00].

**Table 7:** Mean absorbed dose (Gy/GBq) calculated with OLINDA1.1, VoxelMed, VoxelMed^(λ RD)^ and RAYDOSE for kidneys, liver and spleen for patients of the Sample B.

Cases	Kidneys				Liver				Spleen			
	OLINDA1.1	VoxelMed	VoxelMed ^(λ RD)^	RAYDOSE	OLINDA1.1	VoxelMed	VoxelMed ^(λ RD)^	RAYDOSE	OLINDA1.1	VoxelMed	VoxelMed ^(λ RD)^	RAYDOSE
**1**	0.62	0.54	0.54	0.54	0.04	0.03	0.03	0.03	1.11	1.05	1.08	1.04
**2**	0.40	0.36	0.37	0.40	0.23	0.20	0.22	0.22	0.16	0.16	0.16	0.20
**3**	0.87	0.76	0.77	0.78	0.40	0.37	0.38	0.38	0.58	0.47	0.49	0.49
**4**	0.32	0.28	0.29	0.29	0.12	0.11	0.12	0.12	0.22	0.19	0.20	0.19
**5**	0.42	0.36	0.36	0.37	0.04	0.03	0.04	0.04	0.12	0.10	0.12	0.11
**6**	0.52	0.44	0.46	0.46	0.11	0.10	0.11	0.11	0.92	0.83	0.87	0.84
**7**	0.39	0.33	0.34	0.34	0.10	0.08	0.08	0.09	0.46	0.39	0.44	0.43
**8**	0.75	0.66	0.66	0.67	0.02	0.03	0.03	0.03	0.28	0.24	0.27	0.26
**9**	0.36	0.30	0.30	0.34	1.65	1.45	1.56	1.55	0.38	0.31	0.33	0.36
**10**	0.62	0.55	0.56	0.54	0.08	0.07	0.07	0.08	N/A	N/A	N/A	N/A
**11**	0.48	0.42	0.45	0.44	0.34	0.30	0.35	0.33	0.67	0.58	0.90	0.84
**12**	0.66	0.57	0.56	0.57	0.13	0.10	0.11	0.12	0.21	0.17	0.18	0.19
**13**	0.30	0.25	0.26	0.28	0.08	0.07	0.07	0.08	0.34	0.28	0.33	0.34
**14**	0.48	0.42	0.42	0.41	0.06	0.05	0.06	0.06	0.10	0.08	0.09	0.09
**15**	0.40	0.34	0.36	0.36	0.03	0.03	0.03	0.04	0.62	0.54	0.68	0.66
**16**	0.27	0.23	0.25	0.27	0.30	0.27	0.30	0.29	0.52	0.41	0.45	0.45
**17**	0.29	0.25	0.24	0.24	0.09	0.07	0.07	0.07	0.36	0.29	0.28	0.27
**18**	0.51	0.45	0.43	0.44	0.07	0.06	0.07	0.07	0.31	0.24	0.24	0.23
**19**	0.45	0.38	0.40	0.44	1.43	1.20	1.21	1.20	0.83	0.69	0.93	0.95
**20**	0.38	0.47	0.48	0.51	0.11	0.09	0.10	0.11	0.38	0.30	0.33	0.35

## Discussion

In this study we compared the performances of three tools for dosimetry calculations (OLINDA1.1, VoxelMed2.0 and RAYDOSE) with the primary aim to evaluate the influence of the calculation modality on absorbed dose assessment (organ-level based, voxel-level dose kernel convolution based and Monte Carlo simulations based, respectively). The secondary aim was to give some recommendations about the choice of the adequate technique for dosimetry calculation to be implemented in a hospital, in a research centre or in an academic institute (clinical or research purpose; small or large number of patients; clinical trials only or standard procedures). This analysis was performed in standard conditions by acquisition and processing of radioactive phantoms (provided with inserts of specific volume and geometry) and in clinical conditions over a large cohort of patients (a selection of clinical cases taken from a clinical trial in which dosimetry had already been calculated). Clinical conditions are indeed quite distant from and more complicated than the standard conditions achievable in a phantom for several reasons: biological kinetics in place of only physical decay of activity, serial acquisitions of functional images and associated issues related to image registration [43], motion of the patient that creates artefacts in images, irregular shape of volumes of interest, inhomogeneous activity distribution.

Therefore, to consider a large sample of clinical cases was of great interest, since many studies about methods for dosimetry calculation are based on smaller groups of patients [18–20] and in a small group the inter patient variability cannot be properly investigated.

The quantitative inter-comparison between all the three software was performed between the mean values of absorbed doses. In fact, OLINDA provides only mean values, while RAYDOSE and VoxelMed2.0 (voxel-based tools) provide the dose distribution (that can be represented with DVHs) from which the mean dose values can be derived. To compare the techniques of calculation, the relative differences and the correlation between data pairs were evaluated.

For standard conditions, we evaluated discrepancies of calculated absorbed dose in a cylindrical phantom and in differently shaped inserts filled with a homogeneous radioactive solution. Table 5 shows the values of absorbed dose obtained with OLINDA1.1, VoxelMed and RAYDOSE in each of the phantoms. These values are also plotted in Fig 3. Lower values of absorbed dose were generally calculated using OLINDA, in comparison with the dose calculated with other voxel modalities. In the case of the cylindrical phantom, a good agreement was obtained between VoxelMed2.0 and RAYDOSE (discrepancy equal to 5%), while larger difference was observed between VoxelMed2.0 and OLINDA (13%). Absorbed dose map provided by VoxelMed and RAYDOSE showed similar spatial distribution, close values of standard deviation across voxels (around 20%) and analogues slope in DVHs (Fig 4).

To compare the calculation techniques in different conditions of volume and geometry the Geometrical phantoms were acquired. Relative differences in absorbed dose depend on the shape and on the volume of the inserts; smaller is the volume and further from a regular sphere is the shape, more the relative difference is higher. In the Geometrical phantom the toroidal inserts provided the greatest discordance (relative difference with VoxelMed dose ranging from -52% to -41% for OLINDA and from -25% to -16% for RAYDOSE), while in the other inserts differences ranged between [-23%, 0%] for OLINDA1.1 and [-8%, +4%] for RAYDOSE. On one hand, the insert dose calculations in OLINDA were performed using the sphere model, since OLINDA only allows to perform dosimetry calculation for specified models (i.e. organs or spheres). This approximation might explain the huge discrepancies obtained with the voxel-based methods. On the other hand, a reason for the difference between RAYDOSE and VoxelMed is that the latter applies a mask before the convolution, while RAYDOSE does not. This contribution affects calculations in so far as the geometry and the volume of the insert may influence the activity distribution and leave empty spaces around or inside the objects. This effect is especially pronounced, for example, in the case of the toroid. The application of a mask also implies the lack of photon cross irradiation contribution between inserts, which has an impact on dose calculation (contribution around 5% [19]). Discrepancies of calculated doses in the Anthropomorphic phantom were smaller in comparison with the Geometrical phantom. Relative differences around 4% and 11% for RAYDOSE and OLINDA, compared to the VoxelMed dose, were obtained. The larger volume of the inserts and the use of a more appropriate model in OLINDA is likely to have reduced the discrepancies. These results pointed out the tendency to provide higher value of absorbed doses calculated with the RADAR organ dosimetry (OLINDA) method in comparison to voxel-level dosimetry techniques. These differences could be partially due to that OLINDA assumes all electron sources to be locally deposited, while this assumption is not realized using voxel-based approaches. This tendency was shown also by other authors. Kletting et al [18] in their study obtained doses for their software NUKDOS around 1% to 2% lower than values obtained with OLINDA according to their workflow. However, NUKDOS performs voxel-dosimetry based on one SPECT/CT and on a series of planar images to determine the organ pharmacokinetics, making it much closer to organ-level dosimetry approach than VoxelMed. Hippeläinen et al. [19] reported relative differences of 6%, 5%, 0% for left kidney, right kidney and spleen respectively when comparing their dosimetry software HIRD with an analytical dose calculation in the dynamic XCAT phantom study. These values were obtained in closer condition to our study than NUKDOS and can be compared to values around 11% collected in Table 5.

An advantage of voxel-based methods is the ability to calculate DVHs and to show isodose lines, which can assist in treatment optimization [8, 44]. The DVH is already extensively used in EBRT, and it is an important instrument to predict the effect of absorbed dose to tumour and tissues, as supported by [45–47]. With reference to DVHs, VoxelMed and RAYDOSE for geometrical and anatomical inserts provide similar slopes, demonstrating a good accordance for the spatial distribution of the dose calculated with these two techniques (Fig 5). DVHs explain the differences discussed above between the mean values for absorbed doses, by adding the value of the dose distribution analysis.

The differences and the weak points in dose calculations pointed out on phantoms become essential to investigate and to explain the differences in clinical cases. Two different clinical comparisons were performed. For a copious number of patients (Sample A) dosimetry was calculated with OLINDA1.1 (as in clinical trial) and VoxelMed, while for a smaller and independent number of patients (Sample B) all the three software were considered. Similarly to the phantoms, also for patients it was observed the tendency to provide lower value of absorbed dose calculated with voxel-level techniques in comparison with the organ-level technique. In Sample A, VoxelMed calculated dose was 8% lower than OLINDA1.1 calculated dose (for all patients and all organs). This value is smaller than the 11% obtained with phantoms. However, large variability was observed (Fig 6) and the discrepancy was 11% if the absolute percentage difference was considered. The large inter-patient variability is probably due to the inhomogeneity of activity and the use of a set of sequential images. These results were confirmed for patients in Sample B: VoxelMed doses were 13% lower than OLINDA1.1 doses (14% in absolute values) and 8% lower than RAYDOSE doses (9% in absolute values). Similarly, to the phantoms, patient differences were lower and correlations larger (0.79 against 0.97) between VoxelMed and RAYDOSE than OLINDA1.1. Furthermore, correlation was larger with liver than with kidney and spleen in both cases. This is probably due for kidneys to the smaller volume in comparison with liver and for spleen to the breathing artefacts that likely hugely affect the quality of the rigid registration of sequential images (and then of the dose calculation), as reported also by Hippeläinen et al. [19].

Finally, the importance of the activity integration technique in the dose calculation was pointed out: the smallest discrepancies and almost perfect correlation (LC~1) between VoxelMed and RAYDOSE results were obtained when the same decay constant was used.

Compared to the work from the same group previously published [23], the present study provides a very large cohort of patients from the same clinical trial to support data with good statistics. Besides, the new version of VoxelMed (VoxelMed2.0) is run, which is different from the previous version for several points presented in section B.4.1, and also a Monte Carlo based software was considered for comparison. The main drawback of the previous study was to consider VOIs drawn in different workstations for each dosimetry tool, though the set of images was the same. This aspect was considered a source of inaccuracy in the comparison at that time. The average relative difference between VoxelMed and OLINDA in that work was 30% in kidneys in clinical cases, while it decreased down to 14% in this study.

These ameliorations compared to the previous work of the same group and the actual presentation of three different techniques to cover almost all the current possibilities for dosimetry computation allow to delineate the recommendations to the user that has been approaching dosimetry for the first time.

Following the results of the present work, authors can conclude that in dosimetry calculations and in the harmonization process of different dosimetry software there are critical steps that may be summarized as: contouring of volumes of interest; matrices of S values and type of convolution used to calculate absorbed doses; calculation over the whole field or on a restricted region of the 3D image; time activity curve fitting and integral from the first to the last image time point; time activity curve extrapolated from the last time point to infinity; time required for calculations; degree of personalization of the technique.

The use of different settings in these steps may provide very different results: all these steps should be deeply investigated on several real cases before implementing a new home-made or commercial system, based on voxel level or on organ level calculations.

## Conclusion

This study provides valuable confirmation that different results may be obtained with different calculation modalities, especially when using organ-level rather than voxel-level methods.

If a more personalised dosimetry is desired, then a voxel-level method should be preferred. A voxel based calculation allows anyway to calculate a mean absorbed dose value as average of the voxel values inside a volume of interest. The overall time required for computation may be longer for voxel based than for organ based techniques, depending on the software used. The home-made software provides a full customization of the procedure, yet the calculation can be tricky. The commercial systems for a 3D workflow can be safer for the user and easier to use, but the proper customization may be difficult for the user.

Among the voxel based techniques, the MC simulations, though considered the most accurate, may sometimes take too long to calculate dosimetry or may require very powerful computers to run fast. These two characteristics can be more adequate for a research hospital or university, than for exclusive clinical use on a high number of patients. For the latter, an easy to use system (organ or voxel based) could be the best choice until new advanced, fast and practical computation systems are not available on a large scale.

The difference in the choice of the proper calculation system is made also by the need to calculate absorbed dose to organs at risk only, or to organs and tumours. The voxel based techniques should be preferred in all those cases in which tumour absorbed dose is to calculate, especially in case of less extended tumours with irregular shapes far from the spherical geometry.
